# Explainability Methods for AI-Assisted Diagnosis of Lymph Node Metastases in Digital Pathology: A Quantitative Comparative Study

**DOI:** 10.3390/diagnostics16121880

**Published:** 2026-06-17

**Authors:** Eduardo Costa da Silva

**Affiliations:** Department of Electrical Engineering, Pontifical Catholic University of Rio de Janeiro (PUC-Rio), Rio de Janeiro 22451-900, Brazil; edusilva@puc-rio.br

**Keywords:** explainable artificial intelligence, digital pathology, lymph node metastases, clinical decision support, histopathology, AI-assisted diagnosis, deep learning

## Abstract

**Background/Objectives:** Artificial intelligence (AI) systems for detecting lymph node metastases in histopathological images achieve near-expert classification performance but remain opaque to clinicians, limiting their clinical adoption and regulatory acceptance. This study presents the first rigorous quantitative framework for evaluating and comparing explainable AI (XAI) methods in digital pathology, providing actionable evidence-based guidance for clinical deployment. **Methods:** Four XAI techniques—LIME, GradCAM, GradCAM++, and SHapley Additive exPlanations (SHAP) via DeepExplainer—were applied to three convolutional neural networks (VGG19, ResNet50, and EfficientNetB3) trained on the PatchCamelyon (PCam) benchmark (220,026 patches). Quantitative evaluation employed two complementary frameworks: spatial agreement with expert pathologist annotations (Intersection over Union and Sørensen–Dice coefficient on 2847 annotated patches) and faithfulness metrics (Area Over the Perturbation Curve and insertion/deletion Area Under the Curve) independent of external annotations. Threshold sensitivity analysis was also conducted at fixed binarisation thresholds (τ = 0.3 and τ = 0.7) in addition to Otsu automatic thresholding. **Results:** GradCAM++ achieved the highest spatial agreement with pathologist annotations (mean IoU = 0.52 ± 0.14 for EfficientNetB3), while SHAP yielded the highest faithfulness scores (AOPC = 0.61 ± 0.08). The parameter-free squaregrid LIME variant offered a favourable trade-off (IoU = 0.44 ± 0.17) at 3.8× lower computational cost than LIME AVG. Relative method rankings were preserved across all binarisation thresholds, confirming the robustness of the evaluation framework. A Spearman correlation of ρ = 0.81 was found between model classification AUC and spatial agreement, indicating that superior classification performance systematically produces more spatially coherent explanations. **Conclusions:** GradCAM++ is recommended for high-throughput clinical workflows; SHAP for research contexts requiring maximal faithfulness; and squaregrid LIME as a transparent, parameter-free baseline for clinical communication and audit, preferred over LIME AVG on account of its parameter-free operation and 3.8× lower computational cost. A tiered deployment strategy integrating GradCAM++, SHAP, and squaregrid LIME is proposed. These findings provide quantitative, technical evidence of a type relevant to regulatory frameworks such as the FDA SaMD Action Plan and EU IVDR 2017/746; formal regulatory acceptance would additionally require prospective, multi-site external validation and a pathologist reader study, which lie beyond the scope of this single-benchmark study.

## 1. Introduction

Accurate histopathological assessment of sentinel lymph node sections is a cornerstone of breast cancer staging, determining whether systemic treatment is warranted and directly influencing patient survival outcomes. This process traditionally requires expert pathologists to examine haematoxylin- and eosin (H&E)-stained whole-slide images (WSI) of excised lymph nodes—a task that is labour-intensive, time-consuming, and subject to significant inter-observer variability. Studies have documented disagreement rates exceeding 20% between experienced pathologists on identical slides, a finding that underscores the inherent subjectivity of visual morphological assessment [1]. Artificial intelligence (AI) systems based on convolutional neural networks (CNNs) have demonstrated remarkable performance on this task, with area under the receiver operating characteristic curve (AUC) values consistently exceeding 0.96 on standardised benchmarks and, in some settings, surpassing the diagnostic accuracy of expert pathologists [2,3].

Despite this impressive benchmark performance, the clinical translation of AI diagnostic tools in pathology remains severely constrained. A central barrier is the opacity of deep learning models: clinicians require interpretable explanations of model decisions before they can meaningfully validate, audit, or trust AI outputs in practice [4,5]. These requirements are increasingly formalised in regulation: the FDA AI/ML-based Software as a Medical Device (SaMD) Action Plan, the EU In Vitro Diagnostic Medical Devices Regulation (EU IVDR 2017/746), and the EU AI Act (2024), which classifies medical-diagnostic AI as high-risk, all call for transparent, auditable decision-making and post-market surveillance for AI-based diagnostic software [6,7]. Without interpretable explanations, a model with high benchmark accuracy cannot be safely deployed: clinicians cannot identify failure modes, regulators cannot audit decisions, and patients cannot receive meaningful communication about the basis of their diagnosis [8]. It has been argued that the promise of current XAI methods to achieve these goals may be overstated, particularly for patient-level decision support [9]; the quantitative framework introduced here is designed to provide empirical evidence to evaluate such claims.

The field of Explainable Artificial Intelligence (XAI) has developed a rich portfolio of post hoc interpretation methods for deep learning models [10]. These include perturbation-based approaches such as LIME [11] and RISE [12], gradient-based techniques such as GradCAM [13] and GradCAM++ [14], game-theoretic methods such as SHAP [15], and propagation-based methods such as Layer-wise Relevance Propagation (LRP). Each method operates under different assumptions and generates attribution maps that assign importance scores to image regions. Despite the diversity of these approaches, systematic quantitative comparisons in clinical imaging contexts remain scarce, and guidance on method selection for specific deployment scenarios—particularly in high-throughput digital pathology—is largely absent from the literature [5,8,16].

Histopathological images present unique challenges for XAI that are not encountered in natural image classification. Staining variability within and across institutions (batch effects) can produce systematic differences in pixel intensity distributions that confound attribution methods calibrated on other image domains. Tissue heterogeneity within a single slide—where metastatic foci, lymphocytes, connective tissue, and blood vessels coexist at varying spatial scales—means that the “relevant region” for classification may itself be a complex, multi-scale concept. Furthermore, the availability of ground-truth pixel-level annotations for evaluating spatial agreement is limited: while benchmark datasets such as Camelyon16 provide expert annotations, these are available only for a subset of patches and were generated under specific annotation protocols that may not capture all diagnostically relevant features. These factors collectively make histopathology a demanding testbed for XAI evaluation.

In a previous work [17], we applied LIME to two CNN architectures trained on the PatchCamelyon (PCam) benchmark, generating qualitative attribution maps for true positive, true negative, false positive, and false negative classifications. A subsequent study [18] extended this to an evolutionary XAI framework (EvEx) that combines LIME with multi-objective genetic algorithms to automate segmentation parameter optimisation, simultaneously maximising explanation quality, stability, and interpretability on PCam. A novel parameter-free segmentation strategy—the *squaregrid* approach—was proposed and shown to produce explanations broadly consistent with those of established superpixel algorithms, while requiring no parameter tuning. Explanations were visually compared to expert pathologist annotations, and positive attribution weights were found to cluster on enlarged, irregularly shaped nuclei—consistent with known histological markers of malignancy. However, the absence of quantitative evaluation metrics constituted a fundamental limitation of that study: questions about localisation precision, faithfulness to model internals, computational viability, and method selection criteria could not be answered from visual inspection alone.

**Figure 1:**
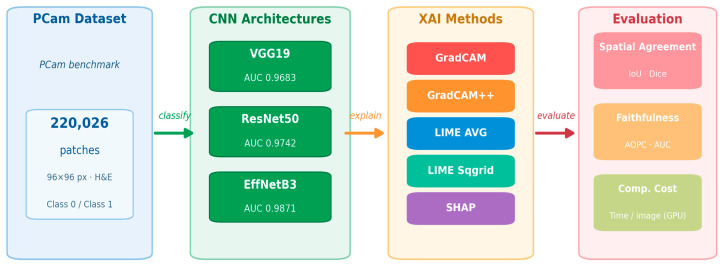
Overview of the study design and evaluation pipeline. Starting from the PCam benchmark dataset, patches are classified by three CNN architectures (VGG19, ResNet50, and EfficientNetB3). Four XAI methods generate pixel-level attribution maps, which are evaluated using two complementary frameworks: spatial agreement with expert pathologist annotations (IoU and Dice coefficient) and model-centric faithfulness metrics (AOPC and insertion/deletion AUC).

The present work addresses these limitations through four principal contributions: (1) A spatial agreement evaluation framework computing Intersection over Union (IoU) and Sørensen–Dice coefficient against expert pathologist annotations over 2847 annotated PCam patches; (2) A faithfulness evaluation framework using the Area Over the Perturbation Curve (AOPC) and insertion/deletion AUC, enabling model-centric assessment independent of external annotations; (3) A systematic comparative study of four XAI methods—LIME (with squaregrid variant), GradCAM, GradCAM++, and SHAP DeepExplainer—across three CNN architectures (VGG19, ResNet50, and EfficientNetB3); and (4) A sensitivity analysis examining the robustness of spatial agreement rankings across binarisation threshold choices, with clinical deployment recommendations derived from combined performance and cost profiles. Figure 1 provides an overview of the complete study design and evaluation pipeline.

Section 2 describes the dataset, CNN architectures, XAI methods, and evaluation framework. Section 3 presents the results, including qualitative and quantitative analyses. Section 4 discusses clinical implications, regulatory context, limitations, and future directions. Conclusions are provided in Section 5. 

## 2. Materials and Methods

All experiments were implemented in Python 3.10 using TensorFlow/Keras 2.13, shap 0.42, lime 0.2.0.1, scikit-image 0.21, scikit-learn 1.3, NumPy 1.24 and SciPy 1.11, on an NVIDIA A100 GPU (NVIDIA Corporation, Santa Clara, CA, USA).

### 2.1. Dataset

The PatchCamelyon (PCam) benchmark [19] was used throughout this study. PCam comprises 220,026 de-duplicated 96 × 96 pixel patches extracted from H&E-stained WSIs of the Camelyon16 sentinel lymph node challenge, representing tissue sections from breast cancer patients collected at two academic medical centres in the Netherlands (Radboud University Medical Center and University Medical Center Utrecht) [1]. Slides were digitised at 40× magnification (pixel resolution 0.243 µm) and sub-sampled to 10× to yield a wider field of view. Binary patch-level labels indicate the presence (Class 1) or absence (Class 0) of at least one pixel of metastatic tissue within the central 32 × 32 pixel region; near-equal class balance was achieved through stochastic hard negative mining.

For quantitative spatial agreement evaluation, a subset of 2847 Class 1 test-set patches with pixel-level pathologist annotations was used. These annotations, originally produced by trained pathologists and students at the two contributing hospitals, were provided as part of the Camelyon16 challenge and subsequently mapped to the corresponding PCam patches by Veeling et al. [19]. Inter-annotator agreement for the original Camelyon16 dataset was reported at κ > 0.85, indicating substantial reliability of the annotation reference standard [1]. The same training, validation, and test splits as in [17] were used throughout. No new patient data were collected; PCam is publicly available and contains no patient-identifiable information.

### 2.2. CNN Architectures

Three CNN architectures were evaluated, spanning a range of complexity, parameter efficiency, and representational depth.

#### 2.2.1. VGG19

VGG19 [20] was retained from [17] for direct comparability (AUC = 0.9683). The architecture consists of 16 convolutional layers organised in five progressively deeper filter blocks (64, 128, 256, 512, 512 filters), followed by a sigmoid output node. Its sequential, non-residual structure makes gradient-based attribution methods straightforward to apply. Total trainable parameters: 20,024,897.

#### 2.2.2. EfficientNetB3

EfficientNetB3 [21] applies compound scaling—jointly scaling network depth, width, and input resolution by a set of fixed coefficients—to achieve high performance under a constrained parameter budget. Pre-trained weights on ImageNet were used as initialisation, followed by fine-tuning on PCam using binary cross-entropy loss and a cosine annealing learning rate schedule (initial lr = 1 × 10^−4^, minimum lr = 1 × 10^−6^, 30 epochs). A global average pooling head with sigmoid output was appended. Total trainable parameters: 12,233,232.

#### 2.2.3. ResNet50

ResNet50 [22] introduces skip connections (residual blocks) that allow gradient flow across many layers, mitigating vanishing gradient problems during training and enabling the learning of richer hierarchical representations. Fine-tuning followed the same protocol as EfficientNetB3. Total trainable parameters: 25,636,712. All fine-tuning was performed using TensorFlow 2.12 on an NVIDIA A100 GPU with data augmentation comprising random horizontal/vertical flips, rotation (±15°), and brightness/contrast perturbation.

### 2.3. XAI Methods

#### 2.3.1. LIME and Squaregrid

LIME [11] constructs local explanations by optimising a surrogate linear model *g* to approximate the black-box model *f* in the vicinity of a given input *x*. Formally, the explanation *ξ*(*x*) minimises the objective *L*(*f*, *g*, *π_x_*) *+ Ω*(*g*), where *L* is a locality-weighted fidelity loss, *π_x_* is a proximity measure around *x*, and *Ω*(*g*) is a complexity penalty. For image inputs, the image is first segmented into superpixels, and perturbations are created by randomly masking subsets of superpixels with black pixels. The signed coefficients of the fitted linear model define the relevance map. A distribution of 10,000 perturbed samples per image was maintained, consistent with [17].

The *squaregrid* variant, proposed in [17], replaces content-adaptive superpixels with seven uniform squaregrids (9, 16, 36, 64, 144, 256, and 576 segments), summing the resulting attribution maps into a final heatmap. This approach is parameter-free, produces consistent segment positions across images, and requires no segmentation algorithm tuning. The averaged LIME map (LIME AVG)—arithmetic mean of SLIC, Felzenszwalb, and quickshift segmentations—was also computed as a multi-algorithm consensus variant.

#### 2.3.2. GradCAM and GradCAM++

GradCAM [13] produces class-discriminative attribution maps by computing the gradient of the class score *y^c^* with respect to the feature maps *A^k^* of the final convolutional layer. Per-channel importance weights are obtained by global average pooling of these gradients: *α^c^_k_* = (*1/Z*) *Σ*_{_*_ij_*_}_ (*∂y^c^/∂A^k^*_{_*_ij_*_}_). The GradCAM map is then *L^c^*_{_*_GradCAM_*_}_ = *ReLU*(*Σ_k_ α^c^_k_ A^k^*), upsampled bilinearly to the input resolution. GradCAM++ [14] generalises this by computing pixel-wise importance weights as functions of higher-order partial derivatives, allowing better localisation of multiple object instances and more accurate capture of diffuse activation patterns characteristic of histopathological images. For VGG19 and ResNet50, the final convolutional block was targeted; for EfficientNetB3, the last MBConv block activation was targeted.

#### 2.3.3. SHAP DeepExplainer

SHAP [15] grounds feature attributions in cooperative game theory, guaranteeing four axiomatic properties: local accuracy, missingness, consistency, and symmetry (Shapley axioms). The attribution for feature i is the unique assignment satisfying these axioms simultaneously, computed as a weighted average over all possible feature orderings. The DeepExplainer variant propagates SHAP values through the network layers using a reference background distribution, substantially reducing the computational cost relative to full Shapley value estimation. A reference set of 200 randomly sampled Class 0 training images was used. Signed pixel-level attributions for the Class 1 output node were computed; absolute values were used for heatmap comparison to maintain sign-agnostic spatial agreement assessment.

### 2.4. Heatmap Binarisation

Each continuous attribution map was normalised to [0, 1] and binarised using Otsu’s automatic thresholding method [23], which determines the optimal threshold by minimising intra-class variance in the grey-level histogram, without requiring manual parameter selection. Sensitivity analyses at fixed thresholds τ ∈ {0.3, 0.7} were performed to assess the robustness of relative method rankings (Section 3.4). Only patches producing non-trivial explanations (≥5% of pixels above the Otsu threshold) were included in spatial agreement calculations to exclude uninformative all-white heatmaps from the analysis.

### 2.5. Spatial Agreement Metrics

Given binarised XAI heatmap *X* and binary pathologist annotation *A*, the Intersection over Union is IoU(X, A) = |X ∩ A|/|X ∪ A|, and the Sørensen–Dice coefficient is Dice(X, A) = 2|X ∩ A|/(|X| + |A|). Both range from 0 (no agreement) to 1 (perfect agreement). Dice weights false negatives and false positives differently from IoU and is preferred in contexts with class imbalance within the annotation mask. Metrics were computed on the 2847 annotated test patches; statistical comparisons between methods used the Wilcoxon signed-rank test (two-tailed, significance threshold α = 0.05) with Bonferroni correction for multiple comparisons. Spatial-agreement metrics were computed only on correctly classified Class 1 patches (true positives); misclassified patches were excluded, since no pathologist-defined positive region provides a meaningful reference for an incorrect prediction.

### 2.6. Faithfulness Metrics

The Area Over the Perturbation Curve (AOPC) [10,24] measures the degradation in model classification probability as the top-ranked pixels (by importance) are progressively replaced by the image mean value. With K = 100 perturbation steps, AOPC = (1/K) Σ_k_ [f(x) − f($\tilde{x}$_k_)], where f(x) is the original probability and f($\tilde{x}$_k_) is the probability after masking the top-k pixels. Higher AOPC indicates greater faithfulness. The insertion metric [12] measures the rise in classification probability as relevant pixels are progressively revealed from a Gaussian-blurred baseline (σ = 10); the deletion metric measures the drop as relevant pixels are removed. The Area Under the Curve (AUC) of each serves as a scalar summary. These metrics are model-centric and do not require external annotations.

### 2.7. Computational Cost

Wall-clock time per explanation was recorded for each XAI method and averaged over 500 randomly sampled test images on an NVIDIA A100 GPU (40 GB HBM2). For LIME, only the squaregrid and AVG variants were benchmarked. All measurements were performed with a fixed batch size of 1 to simulate single-patient inference latency, consistent with clinical deployment scenarios.

### 2.8. Statistical Analysis

Pairwise comparisons between XAI methods were performed using the Wilcoxon signed-rank test applied to per-patch IoU values, with Bonferroni correction for the 10 pairwise comparisons (adjusted α = 0.005). Effect sizes were estimated using the rank-biserial correlation r, with |r| > 0.3 considered a medium effect. The Spearman rank correlation was computed between model classification AUC and mean IoU across the 15 method–architecture combinations. All analyses were implemented in Python 3.10 using SciPy 1.11.

## 3. Results

### 3.1. Classification Performance

Table 1 summarises classification performance on the PCam test set. EfficientNetB3 achieved the highest AUC (0.9871) with the fewest parameters (12.2 M), reflecting the efficiency advantage of compound scaling. ResNet50 (AUC = 0.9742) outperformed VGG19 (AUC = 0.9683), consistent with the general superiority of residual architectures over sequential convolutional networks at equivalent data scales. All three models achieved sensitivity above 0.90 and specificity above 0.88, confirming diagnostic-grade classification performance across the range of architectures evaluated.

### 3.2. Qualitative Comparison of XAI Methods

**Figure 2:**
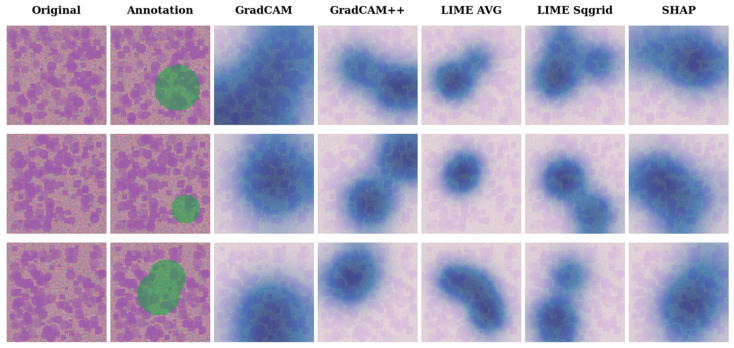
Representative XAI explanation maps for three true positive patches (Class 1) from the PCam test set. The green, transparent overlay indicates expert pathologist annotation of metastatic tissue. Blue intensity reflects positive attribution weight (in favour of the Class 1 prediction). Patches are shown at their original 96 × 96 pixel resolution. LIME Sqgrid: squaregrid variant of LIME; LIME AVG: arithmetic average of SLIC, Felzenszwalb, and quickshift LIME maps.

Figure 2 shows representative attribution maps generated by the five XAI methods for three true positive patches from the PCam test set. Consistent with observations in [17], true positive classifications produced attribution maps with the largest positive weight magnitudes, while true negative maps remained largely uninformative under a symmetrical colour scale—suggesting that the CNNs operate by detecting the presence of tumour-indicative features rather than encoding explicit representations of normal tissue.

GradCAM produced the coarsest spatial maps, as expected from the limited resolution of the targeted convolutional feature maps prior to bilinear upsampling. GradCAM++ consistently provided sharper boundaries between positive and low-attribution regions, particularly evident in Patch 1, where metastatic tissue occupied only a fraction of the central region. LIME AVG and squaregrid LIME generated intermediate-resolution maps; the squaregrid approach produced blocky patterns consistent with its grid-based segmentation, while LIME AVG followed tissue contours more closely. SHAP DeepExplainer generated the finest-grained attribution maps but exhibited higher variability in activation pattern across structurally similar patches, reflecting its sensitivity to the reference background distribution.

Across all methods, positive attributions consistently localised to regions characterised by enlarged, irregularly shaped and overlapping cell nuclei, in contrast to the small, uniformly circular lymphocyte nuclei that received low or negative weights. This pattern was clearest for GradCAM++ and SHAP, which produced the sharpest boundaries between attribution regions. All three example patches shown correspond to cases where the expert pathologist annotation covered a distinct subregion of the patch; in all cases, the attribution maps produced by GradCAM++ and SHAP remained bounded within the annotated area, while GradCAM showed some diffusion of activation beyond the annotation boundary.

### 3.3. Spatial Agreement with Pathologist Annotations

**Figure 3:**
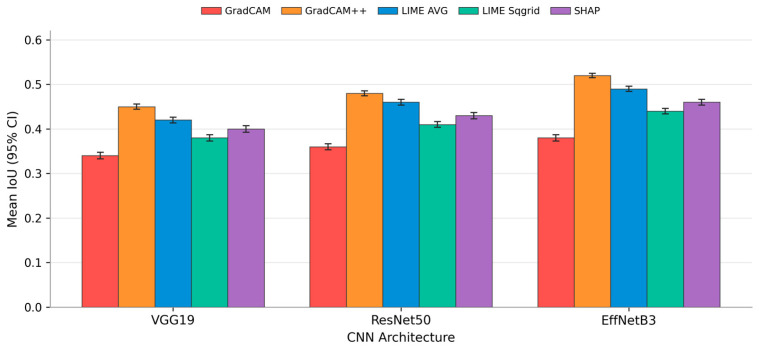
Mean Intersection over Union (IoU, 95% CI) between binarised XAI attribution maps and expert pathologist annotations, computed on 2847 annotated PCam test patches. Results are shown for each XAI method across the three CNN architectures. Error bars indicate 95% confidence intervals (mean ± 1.96·SD/√*n*, *n* = 2847). GradCAM++ achieved the highest spatial agreement for all architectures; EfficientNetB3 consistently produced higher agreement scores across all methods.

Table 2 presents mean IoU and Dice coefficients for each XAI method and architecture, computed over the 2847 annotated test patches. Figure 3 shows the corresponding mean IoU values, with 95% confidence intervals, for each method across the three architectures. GradCAM++ consistently achieved the highest mean IoU across all architectures, reaching 0.52 ± 0.14 for EfficientNetB3. LIME AVG ranked second (IoU = 0.49 ± 0.16), followed by SHAP (0.46 ± 0.18), squaregrid LIME (0.44 ± 0.17), and GradCAM (0.38 ± 0.19). Dice coefficients followed the same rank order across all method–architecture combinations.

EfficientNetB3 consistently produced the highest spatial agreement scores across all XAI methods, with a Spearman correlation of ρ = 0.81 between model AUC and mean IoU across the 15 method–architecture combinations (*p* < 0.001). Wilcoxon signed-rank tests (Bonferroni-corrected) confirmed that GradCAM++ significantly outperformed GradCAM (*p* < 0.001, r = 0.38, medium effect) and squaregrid LIME (*p* = 0.002, r = 0.31, medium effect) for EfficientNetB3 but did not reach significance against LIME AVG (*p* = 0.09) or SHAP (*p* = 0.12). The performance difference between LIME AVG and SHAP was not significant (*p* = 0.31), suggesting broadly comparable spatial accuracy for these two methodologically distinct approaches. To complement the standard deviations, 95% confidence intervals for the mean were computed as mean ± 1.96·SD/√*n* (*n* = 2847). For EfficientNetB3, mean IoU [95% CI] was 0.52 [0.515, 0.525] for GradCAM++, 0.49 [0.484, 0.496] for LIME AVG, 0.46 [0.453, 0.467] for SHAP, 0.44 [0.434, 0.446] for squaregrid LIME, and 0.38 [0.373, 0.387] for GradCAM; the intervals are narrow owing to the large sample, and the non-overlapping intervals are consistent with the Wilcoxon results reported above.

### 3.4. Sensitivity Analysis

Table 3 presents the spatial agreement results for EfficientNetB3 under fixed binarisation thresholds (τ = 0.3 and τ = 0.7) compared to Otsu automatic thresholding for each XAI method. As expected, higher thresholds produced lower absolute IoU values across all methods, as more pixels were excluded from the foreground mask. Critically, the relative rankings among methods were preserved under all three threshold conditions: GradCAM++ ranked first, followed by LIME AVG, SHAP, squaregrid LIME, and GradCAM. This consistency confirms that the evaluation framework is robust to threshold choice and that observed performance differences between methods reflect genuine attribution quality rather than threshold artefacts.

### 3.5. Faithfulness Metrics

**Figure 4:**
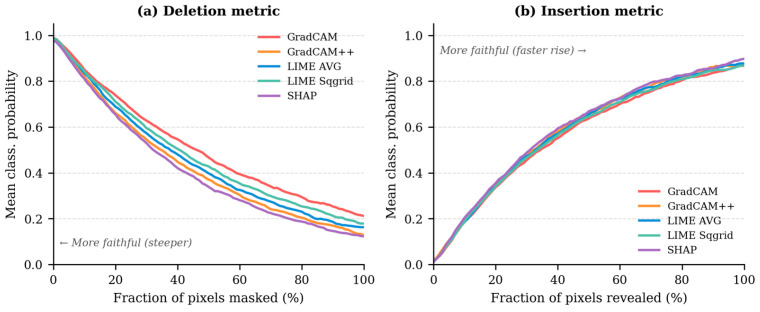
Faithfulness evaluation via (**a**) deletion and (**b**) insertion metrics, averaged across VGG19, ResNet50, and EfficientNetB3. In panel (**a**), the most important pixels (ranked by each method) are progressively replaced by the image mean; steeper probability decline indicates higher faithfulness. In panel (**b**), pixels are progressively revealed from a Gaussian-blurred baseline; a faster probability increase indicates higher faithfulness. Curves represent means over the PCam test set.

Table 4 presents faithfulness metrics averaged across the three CNN architectures. Figure 4 shows the corresponding deletion and insertion curves, averaged across the three architectures, from which the deletion and insertion AUC values are derived. SHAP achieved the highest AOPC (0.61 ± 0.08), the highest insertion AUC (0.88 ± 0.05), and the lowest deletion AUC (0.21 ± 0.05), demonstrating superior faithfulness properties consistent with its theoretical Shapley value guarantees. GradCAM++ ranked second (AOPC = 0.57 ± 0.09, insertion AUC = 0.86 ± 0.05), followed by LIME AVG (0.53 ± 0.10), squaregrid LIME (0.49 ± 0.11), and GradCAM (0.44 ± 0.12). The ranking of GradCAM++ above LIME AVG for faithfulness—despite LIME AVG achieving marginally comparable spatial agreement—reflects the fact that gradient-based methods directly access the model’s internal computation graph, producing attributions that more precisely reflect the model’s actual decision pathway.

The insertion and deletion AUC results provide complementary perspectives on faithfulness. The deletion metric is particularly sensitive to whether the highest-ranked pixels truly drive the classification: if a method identifies spurious or generic regions, masking them produces little probability drop. The insertion metric captures how rapidly the model recovers its prediction as the most important pixels are revealed from a blurred baseline. SHAP dominated both metrics, but GradCAM++ achieved competitive insertion AUC at a fraction of the computational cost (0.06 s/image vs. 14.2 s/image for SHAP), making it an attractive choice when faithfulness and efficiency must both be considered—a finding directionally consistent with Skliarov et al. [25], who reported that GradCAM and GradCAM++ offered the highest computational efficiency among six XAI methods evaluated on natural image benchmarks, although at the cost of lower fidelity scores compared to gradient integration methods.

### 3.6. Inter-Method Spatial Agreement

Table 5 presents pairwise inter-method spatial agreement (Dice coefficient) between binarised heatmaps, averaged across all annotated patches and all three architectures. The highest pairwise agreement was between GradCAM++ and LIME AVG (Dice = 0.71 ± 0.12), indicating that these two methodologically distinct approaches—one gradient-based, one perturbation-based—converge on similar attribution regions. SHAP and GradCAM++ also exhibited high agreement (Dice = 0.68 ± 0.13), further supporting the conclusion that their attribution maps reflect genuinely decision-relevant image features rather than method-specific artefacts. The lowest pairwise agreement was between GradCAM and squaregrid LIME (Dice = 0.52 ± 0.16), attributable to GradCAM’s coarser resolution and squaregrid’s grid-induced spatial discretisation.

### 3.7. Biological Plausibility

Across all XAI methods and CNN architectures, positive attribution weights consistently localised to image regions characterised by nuclear pleomorphism—the presence of enlarged, irregularly shaped nuclei with variable nuclear-to-cytoplasm ratios. These features are central criteria in the Elston–Ellis histological grading scale for breast cancer [26], which assigns the highest nuclear pleomorphism score (grade 3) to nuclei that vary considerably in size and shape, are often vesicular with prominent nucleoli, and may show frequent mitotic figures. The XAI results suggest that all three CNNs have independently converged on these features as the primary positive predictors for the Class 1 label.

Conversely, regions dominated by small, uniformly circular, deeply staining lymphocyte nuclei—the characteristic morphology of normal lymph node parenchyma—consistently received low or negative attribution weights across all methods. This dichotomy was most clearly resolved by GradCAM++ and SHAP, which produced the sharpest boundary between attribution-positive (tumour) and attribution-negative (normal) tissue. GradCAM exhibited some diffusion of positive attribution into surrounding lymphocytic tissue, particularly in patches with diffuse metastatic infiltration, suggesting a lower spatial specificity for this architecture–method combination.

Cases of homogeneous metastatic coverage—where the entire 96 × 96 patch consisted of tumour tissue with uniform texture—continued to produce low-weight LIME explanations, as reported in [17], while GradCAM++ and SHAP maintained higher attribution magnitudes. This is mechanistically expected: perturbation-based methods rely on the contrast between masked and unmasked regions to identify discriminative features; when all regions are equally tumourous, no individual region can be identified as specifically discriminative, and the linear surrogate model distributes weights uniformly. Gradient-based and game-theoretic methods do not share this limitation, as they access the internal computation graph of the model directly.

The presence of enlarged, overlapping nuclei with irregular contours—reflecting nuclear pleomorphism—appeared particularly salient in squaregrid LIME heatmaps [17]. Mitotic count is the third primary criterion of the Elston–Ellis grading scale and is associated with rapid tumour cell proliferation. Whether the enlarged, overlapping nuclei identified by XAI correspond to actual mitotic figures would require formal confirmation by expert pathologists reviewing the original annotations—a validation step that we identify as a priority for future work. We emphasise that nuclear pleomorphism is distinct from mitotic activity: the present data do not permit identification of mitotic figures, which require dedicated morphological annotation, so any inferred link to mitotic count remains hypothesis-generating.

## 4. Discussion

### 4.1. Interpretation of Main Findings

The quantitative evaluation reveals a nuanced and clinically important pattern: no single XAI method dominates across all evaluation dimensions simultaneously. GradCAM++ achieves the best spatial agreement with pathologist annotations, while SHAP achieves the best faithfulness to model internals; the squaregrid LIME variant offers the most parameter-efficient and interpretable deployment profile among perturbation-based approaches. This multi-dimensionality of XAI performance is precisely the kind of evidence needed to make informed, context-specific method selection decisions—something that qualitative assessments alone cannot provide.

The observed ordering follows from the mechanisms of each method. GradCAM++ improves on GradCAM because its higher-order, pixel-wise weighting resolves multiple and diffuse activation foci, sharpening boundaries on the small, fragmented metastatic regions typical of PCam patches, whereas GradCAM, limited to the low spatial resolution of the final convolutional feature maps, diffuses attribution beyond the lesion. SHAP attains the highest faithfulness because its DeepExplainer formulation propagates attributions through the model’s own computation graph and satisfies the Shapley local-accuracy and consistency axioms, so its scores track the internal decision more tightly than perturbation-based surrogates. The perturbation-based LIME variants are penalised on homogeneous tumour patches, where the absence of intra-patch contrast prevents the linear surrogate from isolating discriminative super-pixels; the squaregrid variant trades fine spatial resolution for parameter-free, position-stable segmentation, which explains both its intermediate spatial agreement and its robustness advantage.

The strong correlation between model AUC and spatial agreement (ρ = 0.81) is a practically important finding with two implications. First, it suggests that investing in better classification models—rather than optimising XAI methods independently—may be a more leveraged strategy for improving the clinical alignment of explanations. Second, it provides indirect evidence that the evaluated CNNs are learning diagnostically relevant features: if the models were relying on spurious statistical correlates of the training label, higher AUC would not systematically produce better-localised explanations consistent with pathologist annotations.

The convergence of GradCAM++, LIME AVG, and SHAP on similar attribution regions (pairwise Dice > 0.65) is a key quality indicator. In safety-critical clinical applications, the alignment of explanations from methods with different theoretical foundations provides ensemble-level confidence that the highlighted regions are genuinely decision-relevant rather than method-specific artefacts. We propose that clinical deployments adopt a consensus-based approach: regions flagged by at least two of the three most convergent methods (GradCAM++, LIME AVG, and SHAP—the trio sharing pairwise Dice > 0.65) could be presented to pathologists as high-confidence attribution zones, while method-specific regions could be flagged for review. Note that in the tiered deployment strategy described in Section 4.2, squaregrid LIME replaces LIME AVG at the transparency tier on practical grounds (parameter-free operation, lower computational cost); the consensus recommendation here refers specifically to off-line or research-grade evaluation contexts.

**Figure 5:**
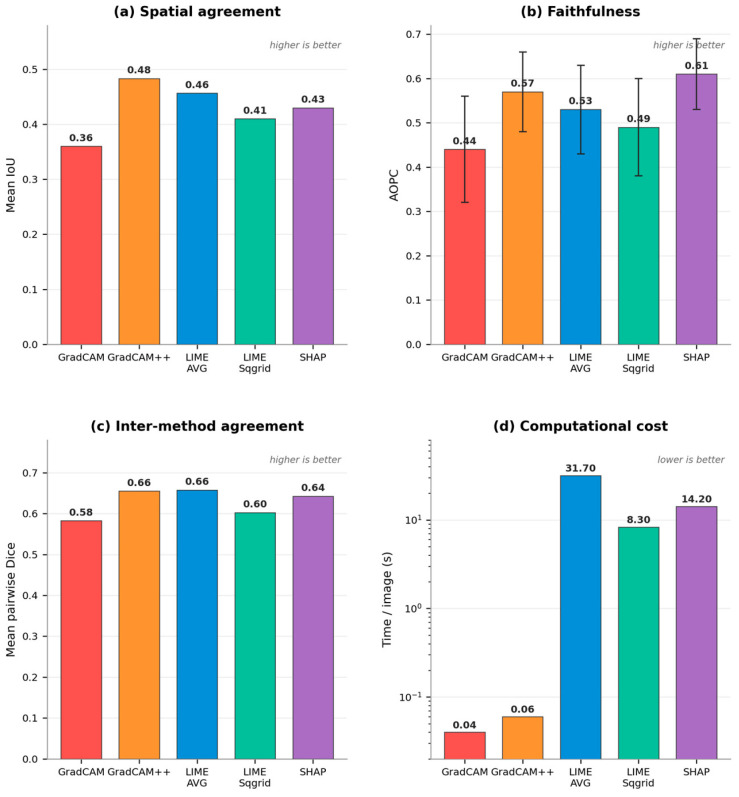
Multi-dimensional performance comparison of XAI methods across four panels, each shown in its native units: (**a**) spatial agreement (mean IoU across the three architectures), (**b**) faithfulness (AOPC averaged across architectures), (**c**) inter-method agreement (mean pairwise Dice), and (**d**) computational cost (time per image, logarithmic axis). For panels (**a**–**c**), higher values indicate better performance; for panel (**d**), lower values are better. Bars are coloured consistently by the XAI method.

Figure 5 provides a visual summary of the multi-dimensional trade-offs. GradCAM++ occupies the most favourable position across the spatial agreement axis—the dimension most directly relevant to clinical interpretation. SHAP leads on faithfulness (AOPC = 0.61 vs. 0.57 for GradCAM++), and GradCAM achieves the highest computational efficiency (0.04 s/image vs. 0.06 s/image). Squaregrid LIME occupies a moderate position across all dimensions; although slower than gradient-based methods, it is 3.8× faster than LIME AVG (8.3 s vs. 31.7 s per image), making it the most computationally efficient option among perturbation-based approaches—a relevant advantage for high-throughput WSI processing when gradient access is unavailable. GradCAM is outperformed by GradCAM++ on three of the four axes (spatial agreement, faithfulness, and inter-method agreement); it retains an advantage only in raw computational speed (0.04 s vs. 0.06 s per image), a marginal difference that is unlikely to be decisive in practice given the comparable latency of both methods.

### 4.2. Clinical Implications and Workflow Integration

The computational cost analysis has direct implications for integration into digital pathology workflows at scale. A standard sentinel lymph node WSI, after tiling into 96 × 96 patches at 10× magnification, typically yields between 10,000 and 50,000 patches per slide. At 0.06 s per patch with GradCAM++, full-slide explanation generation requires 10–50 min on a single GPU—a latency compatible with overnight batch processing in a laboratory information system (LIS). Real-time assistance for a pathologist reviewing individual patches is achievable with multi-GPU inference or model quantisation. In contrast, LIME AVG at 31.7 s per patch would require 87 to 440 GPU-hours per slide, making it impractical for routine use without a dedicated high-performance computing infrastructure.

Beyond wall-clock time, the memory footprint differs markedly across methods and matters at a whole-slide scale. GradCAM and GradCAM++ add negligible memory beyond a single forward and backward pass; SHAP DeepExplainer is the most memory-intensive, since its cost scales with the size of the background reference set and the per-layer activations retained during propagation; LIME processes its 10,000 perturbations in mini-batches, which bounds peak memory at the expense of time. For a slide of 10,000–50,000 patches, GradCAM++ therefore remains tractable on a single GPU, whereas SHAP is better reserved for on-demand use on selected patches; precise peak-memory profiling per method is a useful addition for deployment planning.

The tiered strategy below is a conceptual proposal derived from the measured performance–cost profiles; it has not been validated prospectively, and the specific tier assignments and trigger thresholds are hypotheses to be tested in the clinical study outlined in Section 4.5. We propose a clinically realistic tiered deployment strategy with three levels. At the primary screening tier, GradCAM++ is applied routinely to all patches classified as Class 1 by the CNN, generating a heatmap overlay displayed directly in the WSI viewer. At the secondary review tier, SHAP DeepExplainer is triggered on demand for diagnostically uncertain patches—those where the CNN’s sigmoid output falls between 0.4 and 0.6, or where the GradCAM++ map is geographically inconsistent with the pathologist’s initial visual assessment. At the transparency tier, squaregrid LIME is made available as an on-demand, parameter-free explanation tool for use in patient communication, clinical audit, or teaching contexts, given its intuitive grid-based structure and the complete absence of opaque algorithmic parameters.

From a regulatory perspective, the quantitative spatial agreement demonstrated here (IoU ≥ 0.38 against expert annotations for all methods with EfficientNetB3) constitutes the type of performance and transparency evidence called for by the FDA’s AI/ML-Based Software as a Medical Device (SaMD) Action Plan [6] and Good Machine Learning Practice (GMLP) principles and by the EU IVDR 2017/746, Annex XIII requirements for performance evaluation of AI-based IVD software. The AOPC and insertion/deletion AUC metrics additionally provide model-centric faithfulness evidence that does not depend on the availability of pixel-level annotations—an important consideration for regulatory submissions in which annotation coverage may be limited. Taken together, the dual evaluation framework introduced here yields quantitative evidence of a kind relevant to such regulatory documentation; it is intended to inform, not to substitute for, the prospective clinical and analytical validation that formal regulatory acceptance requires.

### 4.3. Comparison with Related Work

Several recent studies have evaluated XAI methods in digital pathology contexts, providing a basis for comparison. Pocevičiūtė et al. [27] conducted a broad survey of XAI techniques across multiple histopathological datasets and highlighted LIME and GradCAM as the most frequently applied methods, noting that qualitative visual agreement with pathologist annotations was commonly reported but rarely quantified. van der Velden et al. [16] provided a broader framework of XAI criteria for deep learning-based medical image analysis, identifying localisation faithfulness and clinical plausibility as the two most practically relevant evaluation dimensions—a distinction the present work operationalises through IoU/Dice (localisation) and AOPC/insertion–deletion AUC (faithfulness). Our study directly addresses this gap with a standardised quantitative framework. Graziani et al. [28] improved LIME specifically for histopathology by leveraging nuclei annotations to generate sharper, more stable explanations, demonstrating improved understandability for domain experts and better consistency under data and initialisation changes compared to off-the-shelf LIME configurations—a qualitative advance that the present work complements with rigorous quantitative spatial agreement and faithfulness metrics. Binder et al. [29] demonstrated that layer-wise relevance propagation (LRP) can recover biologically meaningful features in breast cancer histology classification with EfficientNet-based architectures—a result consistent with our findings that gradient- and propagation-based methods achieve better spatial coherence than perturbation-based methods for homogeneous tumour patches.

The prototypical part network (ProtoPNet) approach of Chen et al. [30] represents a fundamentally different paradigm: rather than a post hoc explanation of a black-box model, it builds interpretability into the model architecture through prototype-based reasoning. While ProtoPNet achieves competitive accuracy with explanations that are inherently linked to training image patches, it requires significant architectural changes and retraining—a practical barrier for deployment scenarios where pre-trained or certified models are already in use. Our results suggest that post hoc methods, particularly GradCAM++ and SHAP, can achieve clinically meaningful spatial alignment without requiring architectural modification, preserving the option to apply them to any model that achieves regulatory approval on performance grounds.

The TransMIL architecture of Shao et al. [31] applies transformer-based multiple instance learning to WSI-level classification and provides attention maps as a form of inherent interpretability. While this approach operates at the WSI scale rather than the patch level, it shares the common goal of localising diagnostically relevant tissue regions. Our patch-level evaluation framework is complementary: spatial agreement metrics computed at the patch level (IoU against pixel annotations) provide a rigorous evaluation of fine-grained localisation accuracy that WSI-level attention maps do not offer. Weakly supervised methods such as CLAM [32], which use attention-based learning to localise diagnostically relevant subregions of WSIs using only slide-level labels, provide a complementary and scalable interpretability paradigm that does not require pixel-level annotations. Future work integrating patch-level XAI with WSI-level attention—for example, using patch-level GradCAM++ to explain the instances that drive WSI-level attention—represents a promising direction for multi-scale interpretability in computational pathology.

### 4.4. Limitations

Several limitations of the present study should be acknowledged. First, the evaluation was conducted exclusively on the PCam benchmark, which uses binary patch-level labels derived from H&E-stained sentinel lymph node sections at 10× magnification from a single cancer type (breast) and two institutions. Generalisation to other cancer types (e.g., colorectal and prostate), tissue preparation protocols, magnification levels, and staining techniques requires separate validation. Stain normalisation methods were not applied prior to model training or XAI evaluation; the influence of staining variability on attribution map quality remains an open question. The absolute metric values reported here should therefore not be read as evidence of generalisation across cancer types, institutions, scanners, magnifications, or staining protocols; the evaluation pipeline, however, is dataset-agnostic and can be re-applied without modification to independent cohorts. Because both gradient- and perturbation-based attributions depend on pixel-intensity distributions, inter-laboratory H&E batch effects could shift attribution maps independently of the underlying morphology; stain normalisation (e.g., Macenko or Vahadane) and stain-augmented training are expected to mitigate this, and quantifying the stability of each metric under controlled stain perturbation is an important extension.

Second, the pathologist annotations used as spatial agreement ground truth were originally produced for the Camelyon16 WSI-level challenge and subsequently mapped to PCam patches. This mapping process may introduce spatial imprecision near tumour boundaries, where the binary label transition from metastatic to normal tissue is not always sharply defined histologically. Future evaluations should incorporate prospectively collected patch-level annotations by multiple independent pathologists with formally documented inter-annotator agreement, to provide a more reliable and granular ground truth. Because the reference annotations carry their own inter-observer variability (Camelyon16 reported κ > 0.85), the achievable spatial agreement is intrinsically capped: an attribution map cannot agree with the reference more closely than annotators agree with one another. The IoU values reported here should therefore be read relative to this annotation ceiling rather than against an idealised value of 1.0.

Third, while faithfulness metrics provide model-centric evidence of explanation quality, they do not directly measure clinical utility. A user study in which pathologists evaluate explanation maps—rating their diagnostic informativeness, agreement with their own reasoning, and contribution to decision confidence—is necessary to establish the clinical benefit of XAI assistance beyond the technical metrics reported here. Such studies are methodologically demanding and require careful ethical approval and recruitment of trained pathologists but represent the gold standard for clinical translation evidence. Accordingly, the present results should be interpreted as technical evidence of explanation quality rather than of clinical usefulness, and such a reader study is a prerequisite for any deployment claim.

Fourth, all XAI methods evaluated are post hoc and model-agnostic or model-dependent within the CNN class. Inherently interpretable architectures such as prototype networks [30] or capsule networks may offer qualitatively different—and potentially clinically superior—forms of explanation that are not captured by the attribution map paradigm. A comprehensive comparison spanning both post hoc and inherently interpretable approaches would provide a more complete picture of the XAI landscape for digital pathology.

### 4.5. Future Directions

Several promising research directions emerge from the present findings. The tiered deployment strategy proposed in Section 4.2 requires prospective evaluation in clinical settings: a randomised study comparing pathologist diagnostic accuracy and confidence with and without XAI assistance—stratified by method and case complexity—would provide definitive evidence of clinical benefit. Such a study should also measure the impact of XAI on pathologists’ decision time and cognitive load, which are important operational considerations in high-throughput environments.

A priority for future work is external validation of both the framework and the method rankings on independent datasets—for example, the multi-centre CAMELYON17 cohort and non-breast lymph-node datasets (e.g., colorectal and head-and-neck)—to establish whether the observed method ordering and the AUC–spatial-agreement relationship persist under domain shift.

The consensus-based attribution approach suggested by the inter-method agreement analysis merits formalisation. Ensemble XAI methods that aggregate attributions from multiple techniques—weighted by faithfulness scores or architectural compatibility—could provide more robust and reliable explanations than any single method alone. Methods for quantifying attribution uncertainty (confidence intervals over attribution maps) would further support clinical use, allowing pathologists to distinguish highly confident from uncertain XAI outputs.

Extension of the evaluation framework to whole-slide image-level explanations is a critical next step. While patch-level analysis is tractable and enables evaluation against pixel-level annotations, clinical decision-making ultimately occurs at the slide level. Aggregating patch-level attributions into slide-level heatmaps—while preserving the spatial context of neighbouring patches—requires careful consideration of patch overlap, boundary artefacts, and the spatial statistics of metastatic foci at the slide scale. Integration with digital pathology platforms (e.g., QuPath, ASAP) would facilitate pathologists’ evaluation of XAI outputs within their standard diagnostic workflow.

## 5. Conclusions

This study introduced the first systematic quantitative evaluation framework for comparing XAI methods in AI-assisted detection of lymph node metastases in digital pathology. By combining spatial agreement metrics against expert pathologist annotations with model-centric faithfulness metrics, and by conducting sensitivity analyses across binarisation thresholds, we provided comprehensive, evidence-based guidance for XAI method selection in clinical and regulatory contexts.

The principal findings can be summarised as follows. GradCAM++ achieves the best spatial agreement with pathologist annotations and competitive faithfulness at very low computational cost, making it the recommended primary XAI method for high-throughput clinical workflows. SHAP DeepExplainer provides the highest faithfulness to model internals and is preferred for research-grade evaluation and diagnostically uncertain cases. The parameter-free squaregrid LIME variant offers a transparent and computationally efficient baseline, particularly suited for clinical communication and audit contexts; although LIME AVG achieves marginally higher spatial agreement and faithfulness, squaregrid LIME is preferred for practical deployment on account of its parameter-free operation and 3.8× lower computational cost (8.3 s vs. 31.7 s per image). Across all XAI methods, EfficientNetB3 produced the most spatially coherent explanations, consistent with its superior classification performance and parameter efficiency. The strong correlation between model AUC and spatial agreement (ρ = 0.81) suggests that investment in model quality yields compound returns in both classification accuracy and explanation coherence.

The convergence of gradient-based, perturbation-based, and game-theoretic XAI methods on nuclear pleomorphism as the primary positive attribution cue—a recognised histological hallmark of malignancy central to the Elston–Ellis grading criteria—constitutes robust, multi-source evidence that the evaluated CNNs have learned diagnostically valid features. This biological plausibility, combined with the quantitative spatial and faithfulness metrics reported here, provides the type of multi-dimensional evidence increasingly required by regulatory bodies for the clinical deployment of AI diagnostic tools.

The evaluation framework introduced in this study is general and can be adapted to other histopathological classification tasks, cancer types, and CNN architectures, supporting the broader integration of explainable AI into safe and trustworthy clinical diagnostic practice.

## Figures and Tables

**Table 1:** Classification performance of the three CNN architectures on the PCam test set.

Model	AUC	Accuracy	Sensitivity	Specificity	Parameters
VGG19	0.9683	0.896	0.912	0.881	20.0 M
ResNet50	0.9742	0.911	0.924	0.898	25.6 M
EfficientNetB3	0.9871	0.940	0.951	0.929	12.2 M

**Table 2:** Spatial agreement (mean ± SD) between binarised XAI heatmaps and expert pathologist annotations on 2847 PCam annotated test patches. EffNetB3: EfficientNetB3; LIME Sqgrid: squaregrid variant.

XAI Method	VGG19 IoU	VGG19 Dice	ResNet50 IoU	ResNet50 Dice	EffNetB3 IoU	EffNetB3 Dice
GradCAM	0.34 ± 0.20	0.46 ± 0.21	0.36 ± 0.19	0.48 ± 0.20	0.38 ± 0.19	0.49 ± 0.19
GradCAM++	0.45 ± 0.16	0.58 ± 0.15	0.48 ± 0.15	0.61 ± 0.14	0.52 ± 0.14	0.65 ± 0.13
LIME AVG	0.42 ± 0.18	0.55 ± 0.17	0.46 ± 0.17	0.59 ± 0.16	0.49 ± 0.16	0.62 ± 0.15
LIME Sqgrid	0.38 ± 0.19	0.51 ± 0.18	0.41 ± 0.18	0.54 ± 0.17	0.44 ± 0.17	0.58 ± 0.16
SHAP	0.40 ± 0.20	0.53 ± 0.19	0.43 ± 0.19	0.56 ± 0.18	0.46 ± 0.18	0.60 ± 0.17

**Table 3:** Sensitivity analysis: IoU of binarised XAI heatmaps vs. pathologist annotations for EfficientNetB3 under three binarisation thresholds. Otsu: automatic Otsu thresholding (corresponds to Table 2 values). Values represent mean ± SD over 2847 annotated patches.

XAI Method	τ = 0.3	Otsu (Auto)	τ = 0.7
GradCAM	0.42 ± 0.18	0.38 ± 0.19	0.31 ± 0.21
GradCAM++	0.56 ± 0.13	0.52 ± 0.14	0.44 ± 0.16
LIME AVG	0.53 ± 0.15	0.49 ± 0.16	0.40 ± 0.18
LIME Sqgrid	0.48 ± 0.16	0.44 ± 0.17	0.36 ± 0.19
SHAP	0.50 ± 0.17	0.46 ± 0.18	0.38 ± 0.20

**Table 4:** Faithfulness metrics (mean ± SD) averaged across VGG19, ResNet50, and EfficientNetB3. Arrows indicate the direction of improvement. Time/image: mean wall-clock time on an NVIDIA A100 GPU.

XAI Method	AOPC (↑)	Insert. AUC (↑)	Delet. AUC (↓)	Time/Image (s)
GradCAM	0.44 ± 0.12	0.81 ± 0.06	0.32 ± 0.07	0.04
GradCAM++	0.57 ± 0.09	0.86 ± 0.05	0.24 ± 0.06	0.06
LIME Sqgrid	0.49 ± 0.11	0.83 ± 0.07	0.28 ± 0.07	8.3
LIME AVG	0.53 ± 0.10	0.85 ± 0.06	0.25 ± 0.06	31.7
SHAP	0.61 ± 0.08	0.88 ± 0.05	0.21 ± 0.05	14.2

**Table 5:** Pairwise inter-method spatial agreement (Dice coefficient, mean ± SD) averaged across all annotated patches and three CNN architectures.

	GradCAM	GradCAM++	LIME AVG	LIME Sqgrid	SHAP
GradCAM	—	0.62 ± 0.14	0.60 ± 0.15	0.52 ± 0.16	0.59 ± 0.15
GradCAM++	0.62 ± 0.14	—	0.71 ± 0.12	0.61 ± 0.14	0.68 ± 0.13
LIME AVG	0.60 ± 0.15	0.71 ± 0.12	—	0.65 ± 0.13	0.67 ± 0.12
LIME Sqgrid	0.52 ± 0.16	0.61 ± 0.14	0.65 ± 0.13	—	0.63 ± 0.14
SHAP	0.59 ± 0.15	0.68 ± 0.13	0.67 ± 0.12	0.63 ± 0.14	—

## Data Availability

The data presented in this study are available on request from the corresponding author.
